# EAPB0503: An Imiquimod analog with potent *in vitro* activity against cutaneous leishmaniasis caused by *Leishmania major* and *Leishmania tropica*

**DOI:** 10.1371/journal.pntd.0006854

**Published:** 2018-11-21

**Authors:** Rana El Hajj, Hanady Bou Youness, Laurence Lachaud, Patrick Bastien, Carine Masquefa, Pierre-Antoine Bonnet, Hiba El Hajj, Ibrahim Khalifeh

**Affiliations:** 1 Department of Pathology and Laboratory Medicine, American University of Beirut, Beirut, Lebanon; 2 Centre Hospitalo-Universitaire, Université de Montpellier, Montpellier, France; 3 Faculté de Pharmacie, Université de Montpellier, Montpellier, France; Pasteur Institute of Iran, ISLAMIC REPUBLIC OF IRAN

## Abstract

Cutaneous Leishmaniasis (CL) is a parasitic infection classified by the WHO as one of the most uncontrolled spreading neglected diseases. Syria is endemic for *Leishmania tropica* and *Leishmania major*, causing CL in the Eastern Mediterranean. The large-scale displacement of Syrian refugees exacerbated the spread of CL into neighboring countries. Therapeutic interventions against CL include local, systemic and physical treatments. The high risk for drug-resistance to current treatments stresses the need for new therapies. Imiquimod is an immunomodulatory drug with a tested efficacy against *L*. *major* species. Yet, Imiquimod efficacy against *L*. *tropica* and the molecular mechanisms dictating its potency are still underexplored. In this study, we characterized the effect of Imiquimod against *L*. *tropica and L*. *major*, and characterized the molecular mechanisms dictating its anti-leishmanial efficacy against both strains. We also investigated the potency and molecular mechanisms of an Imiquimod analog, EAPB0503, against these two strains. We have tested the effect of Imiquimod and EAPB0503 on macrophages infected with either *L*. *major*, *L*. *tropica* strains, or patient-derived freshly isolated *L*. *tropica* parasites. The anti-amastigote activity of either drugs was assessed by quantitative real time PCR (RT-PCR) using kinetoplast specific primers, confocal microscopy using the Glycoprotein 63 (Gp63) *Leishmania* amastigote antibody or by histology staining. The mechanism of action of either drugs on the canonical nuclear factor kappa- B (NF-κB) pathway was determined by western blot, and confocal microscopy. The immune production of cytokines upon treatment of infected macrophages with either drugs was assessed by ELISA. Both drugs reduced amastigote replication. EAPB0503 proved more potent, particularly on the wild type *L*. *tropica* amastigotes. Toll-Like Receptor-7 was upregulated, mainly by Imiquimod, and to a lesser extent by EAPB0503. Both drugs activated the NF-κB canonical pathway triggering an immune response and i-NOS upregulation in infected macrophages. Our findings establish Imiquimod as a strong candidate for treating *L*. *tropica* and show the higher potency of its analog EAPB0503 against CL.

## Author summary

Cutaneous Leishmaniasis (CL) is a parasitic infection caused by *Leishmania (L*.*)* parasites. In the Old World and the Near East, CL is mainly caused by *L*. *major* and *L*. *tropica*. The ongoing Syrian war and the resulting massive population displacement led to an alarming increase in the incidence of CL, in Syria and its surrounding countries. Current therapies against CL lead to partial or complete cure in *L*. *major* infections but are less effective against *L*. *tropica*. These therapies associate with several limitations, including patients’age, immune system, repetitive painful injections, high cost, poor availability, and mainly systemic toxicity. Therefore, it is of high interest to seek for novel drugs against CL. We assessed the activity of an immunomodulatory drug and its analog against *L*. *major* and *L*. *tropica* parasites and showed their potency. Importantly, the analog proved more efficient against the wild type *L*. *tropica* strain. These results highlight the promising efficacy of immuno-modulatory drugs against CL.

## Introduction

Cutaneous leishmaniasis (CL) is caused by *Leishmania* parasite and is classified by the World Health Organization (WHO) as one of the most common neglected tropical diseases [1]. During the past decade, an alarming increase in the incidence of CL was documented, ranging from 2.1 million cases in 2002, to approximately 4 million cases in 2015 [2]. In the Eastern Mediterranean, *Leishmania tropica* and *Leishmania major* cause CL [3]. In Syria, the prevalence lately doubled due to chronic conflicts [4]. The displacement of Syrian refugees to the neighboring countries, including under-endemic ones like Lebanon, promoted the dissemination of this infection [5].

CL treatment varies among patients [6], and include local, systemic and physical approaches [7]. Meglumine antimoniate (Glucantime) is widely used [8], but yet presents with many disadvantages such as the painful intra-lesional injections to be repeatedly injected in each lesion, on a weekly basis and for up to 8 weeks [9]. An intramuscular injection of Glucantime was proposed to overcome this painful process, however it was associated with high hepatic and cardiac toxicity [10]. Imiquimod is an FDA approved imidazoquinoxaline against skin infections, with great anti-viral/anti-tumor activities [11]. Imiquimod proved potent in CL treatment [12, 13]. It was used in combination with systemic antimonials [14], and presented with cure rates exceeding 90% in refractory patients [15]. Accordingly, it was introduced by the WHO to the guidelines of CL treatment [16].

Among several synthesized Imiquimod analogs [11], EAPB0503 (1-(3-methoxyphenyl)-N-methylimidazo[1,2-a]quinoxalin-4-amine) exhibited higher potency than Imiquimod in several cancer models [17,18,19]. This study addressed the effect of Imiquimod and its analog, EAPB0503, in the context of CL, against amastigote stages of *L*. *tropica* and *L*. *major* parasites. The mechanism of action as well as the elicited immune response were also investigated. This work gives a better insight about the effect of immunomodulatory drugs derivatives on CL, and opens horizons for new and promising treatment paradigm.

## Results

### EAPB0503 exhibited a higher effect on *L*. *tropica* and *L*. *major* amastigotes replication

**Fig 1:**
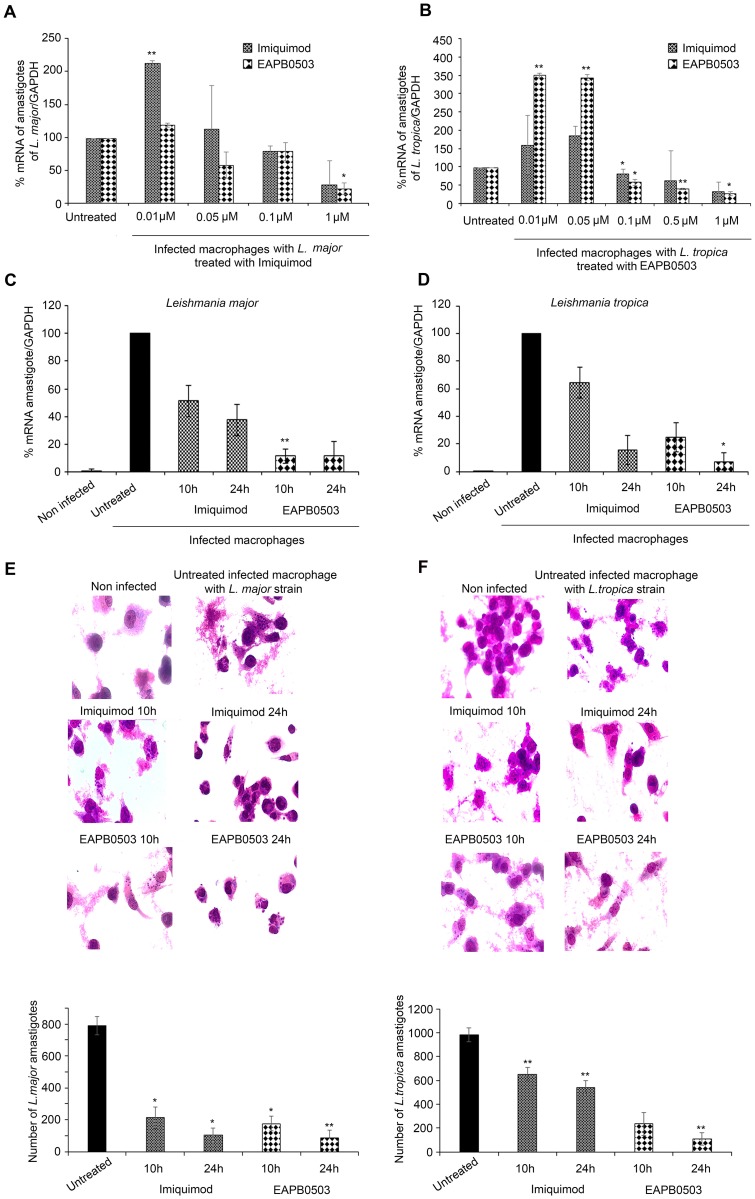
EAPB0503 exhibits a higher efficacy on *L*. *tropica* amastigotes replication. Real-time quantitative PCR detection of infected macrophages with *L*. *major* or *L*. *tropica* amastigotes treated with different concentrations of Imiquimod or EAPB0503 (A, B). RT-PCR detection of infected macrophages with *L*. *major* (C) or *L*. *tropica* (D) amastigotes treated with 0.1 μM of Imiquimod or EAPB0503 for 10 and 24h. Briefly, differentiated and activated THP-1 into macrophages were infected with *L*. *major* or *L*. *tropica* at the ratio of 5 parasites/cell for 24h. Treatment with 0.1, 0.5, 1 or 10 μM of Imiquimod or its analog EAPB0503 was performed for 24h (A-D). Treatment with 0.1μM of Imiquimod or EAPB0503 was performed for 10 and 24h (C, D). The results are shown as percentage of untreated infected macrophages. Amastigote transcripts were evaluated by Syber green RT-PCR using kinetoplast specific primers and their percentage of expression was normalized to GAPDH. Results are expressed as percentage of untreated control (±) SD and are representative of at least three independent experiments. Giemsa staining on untreated or treated macrophages infected with amastigotes of *L*. *major* (E) and *L*. *tropica* (F) strains. Treatment with 0.1 μM of Imiquimod or EAPB0503 was performed for 10 or 24h. The results depict one representative of three independent experiments. The t-test was performed to validate significance. *, ** and *** indicate p values ≤ 0.05; 0.01 and 0.001, respectively. P-values less than 0.05 were considered significant.

To compare the effect of Imiquimod and EAPB0503 on *L*. *major* amastigotes, macrophages were infected at the ratio of 5 parasites per cell. Treatment was performed with different concentrations of either drugs for 24 hours. Amastigotes replication was evaluated by real time PCR, using kinetoplast specific primers. Starting the concentration of 0.1 μM, *L*. *major* amastigotes transcription levels decreased in a concentration-dependent manner following treatment with either drugs, and leading to 80% inhibition of parasite replication at the concentration of 1μM (Fig 1A).

*L*. *tropica*, the most endemic species causing anthroponotic CL (ACL) in the Middle East area [7], showed that both drugs exert an anti-amastigote activity in a concentration dependent manner. Strikingly, a concentration of 0.1 μM was obtained upon treatment with EAPB0503 as compared to 1μM of Imiquimod (10 folds higher concentration) (Fig 1B). This decrease in amastigotes was also more prominent at 0.5 and 1 μM of EAPB0503, compared to the same doses of Imiquimod (Fig 1B). No effect was observed using the vehicle alone (S1 Fig). This promising data clearly shows a different response of leishmanial strains to treatment with either drugs, and a better response obtained upon treatment of *L*. *tropica* strain with EAPB0503.

### EAPB0503 inhibited amastigote replication as early as 10h post-treatment

Based on our concentration screening results, we chose the optimal concentration of 0.1 μM for further analysis. We examined the effect of this concentration at an earlier time point of 10h. Imiquimod induced a decrease in *L*. *major* amastigotes replication by 50% at 10h post-treatment, and by 65% at 24h post-treatment (Fig 1C). More interestingly, EAPB0503 showed a more prominent decrease of amastigotes expression at 10h or 24h post-treatment, where only 10% of amastigote transcripts were detected by RT-PCR (5 folds less than Imiquimod) (Fig 1C).

Imiquimod reduced *L*. *tropica* amastigotes transcription levels to around 60% at 10h post-treatment and to around 20% at 24h post-treatment (Fig 1D). Interestingly, EAPB0503 reduced amastigotes transcript levels to 30% (almost 2 folds less than Imiquimod) at 10h post-treatment and to 10% (around 2 folds less than Imiquimod) at 24h post-treatment (Fig 1D).

We then assessed the effect of both drugs on amastigotes of *L*. *major* and *L*. *tropica* strains histologically. Consistent with the transcript results, both drugs had a leishmanicidal effect on both strains (Fig 1E and 1F). Whilst Imiquimod displayed similar results against *L*. *major* at both time points (Fig 1E), EAPB0503 was more potent against *L*. *tropica* strain (Fig 1F). Altogether, these data show that EAPB0503 acts at the low dose of 0.1 μM and as early as 10h, when compared to its parental compound Imiquimod.

### Imiquimod triggered an increase of TLR-7 expression in *L*. *tropica* infected macrophages

**Fig 2:**
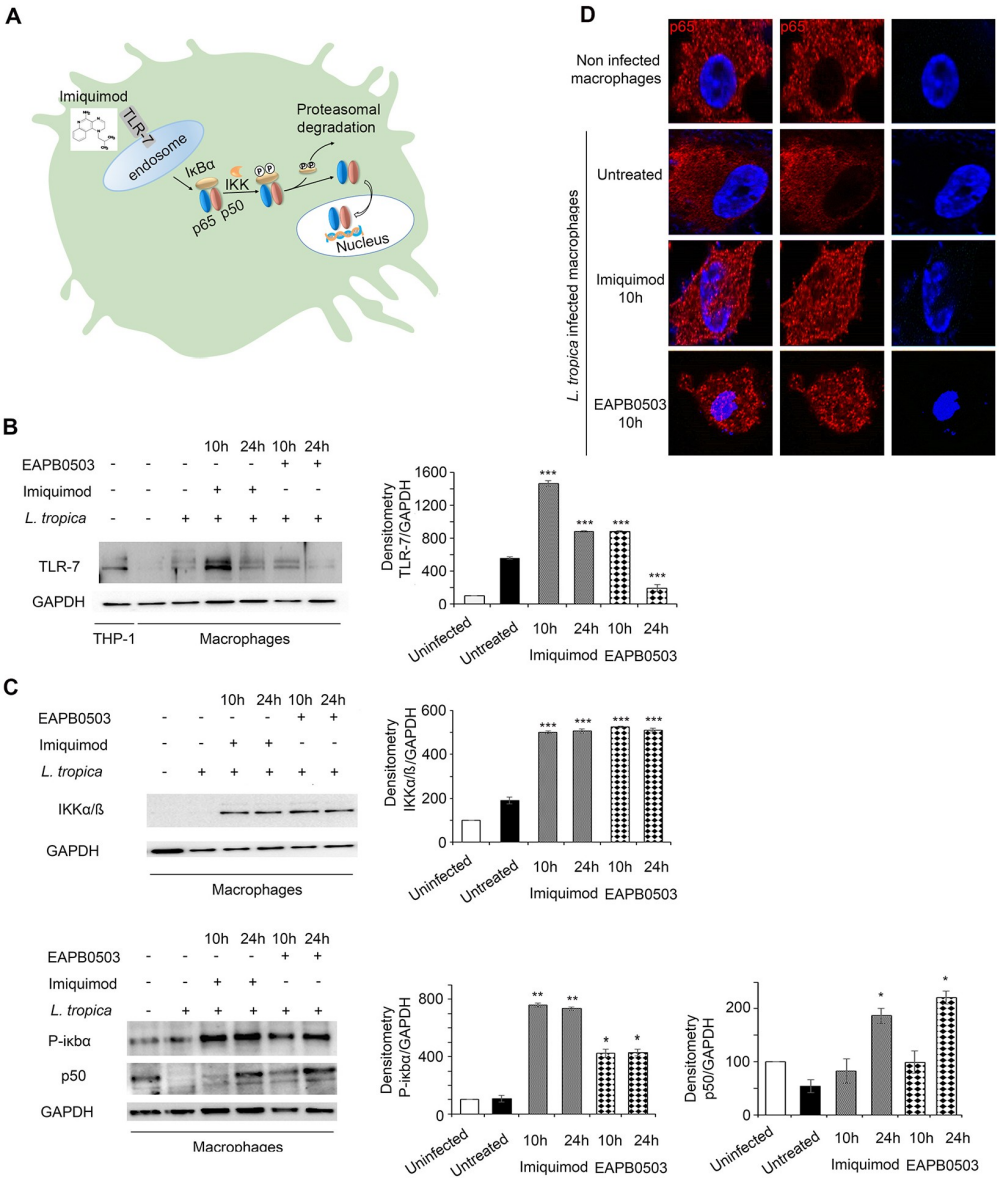
Imiquimod triggers an increase of TLR-7 expression in *L*. *tropica* infected macrophages leading to the canonical NF-κB pathway activation. Schematic representation of the mode of action of Imiquimod through TLR-7 immuno-modulation and its subsequent downstream NF-κB activation (A). Western blot analysis for TLR-7 (B), IKKα/β, P-IκBα and P50 (C) in *L*. *tropica* infected macrophages treated with 0.1 μM of Imiquimod or EAPB0503 for 10 and 24h. The results depict one representative experiment among three independent ones. Densitometry was performed using Image Lab software (Biorad). Results shown represent the average of quantification of three independent experiments. (D) Confocal microscopy on *L*. *tropica* infected macrophages treated with 0.1 μM of Imiquimod or EAPB0503 for 10h. The NF-κB p65 subunit was stained with an anti-p65 antibody (red), and nuclei were stained with Hoechst 33342 (blue). The results depict one representative experiment among three independent ones.

Imiquimod belongs to the class of Toll-like receptor (TLR) agonists with high affinity to TLR7, commonly involved in pathogen recognition (Fig 2A) [14, 20]. We investigated the molecular mechanisms underlying the potency of Imiquimod and its analog EAPB0503 against *Leishmania* amastigotes. We focused on *L*. *tropica* and adopted the concentration of 0.1 μM at both time points 10 and 24h post-treatment. Our results showed that TLR7 protein levels increased after treatment with either drugs, in comparison to uninfected or untreated infected macrophages (Fig 2B). Same results were obtained on *L*. *major* infected macrophages upon treatment with either drugs (S2 Fig 2). In case of *L*. *tropica* and consistent with published data, the upregulation was maximal upon treatment with Imiquimod for 10h (Fig 2B). EAPB0503 induced a higher protein expression of TLR7. Nonetheless, the highest induction of TLR7 was obtained upon treatment with Imiquimod. Our results confirm the mechanism of action of Imiquimod *via* this receptor in the context of CL. The lower expression of TLR7 upon treatment with EAPB0503, seemingly shows a potential mode of action through a different TLR.

### Imiquimod and EAPB0503 induced the canonical NF-κB pathway activation

Following recognition of pathogens, TLRs trigger the NF-κB pathway activation (Fig 2A) [21] inducing immune inflammatory responses [22]. Imiquimod activates the canonical NF-κB pathway (Fig 2A) [23]. We explored this pathway in the context of CL. Western blot analysis clearly showed an activation of the multimeric IKK complex (IKKα/IKKβ) after 10 or 24h treatment with either drugs (Fig 2C). Furthermore, an induction of the phosphorylated form of the IκBα at both time points was obtained, presumably leading to its degradation (Fig 2C). We then examined whether this NF-κB activation involves the canonical pathway. Our results demonstrate that the p50 subunit was upregulated especially upon 24h post-treatment with either drugs (Fig 2C). This led to the nuclear translocation of p65 (Fig 2D), which represents the active NF-κB subunit, and known to activate immune response genes. Collectively these results showed that both Imiquimod and EAPB0503 inhibit amastigote replication *via* activation of the canonical NF-κB pathway.

### Imiquimod and EAPB0503 induced NF-κB mediated macrophage immune response

**Fig 3:**
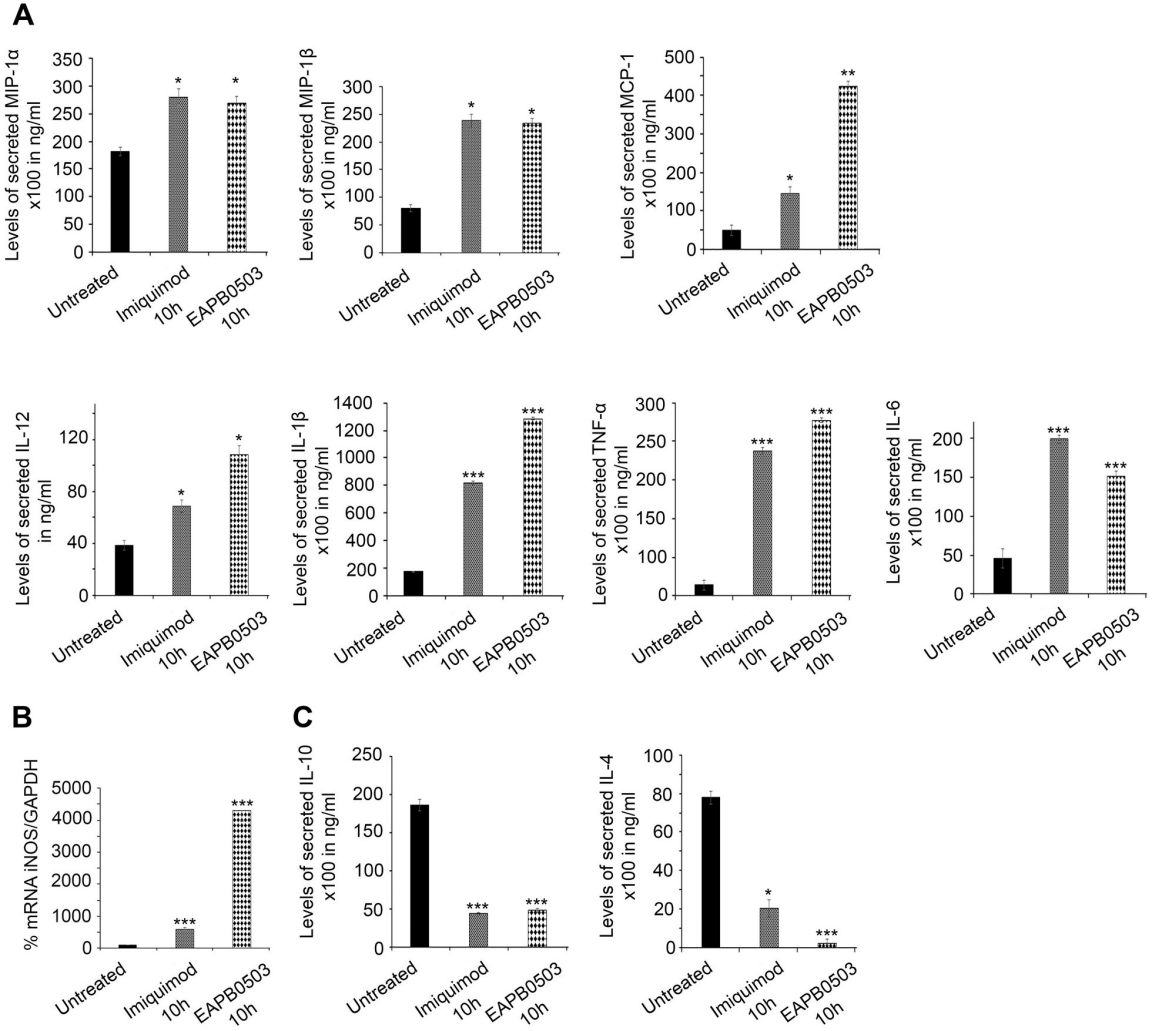
Imiquimod and EAPB0503 induce NF-κB mediated macrophage immune response. (A) ELISA showing the secretion level of the pro-inflammatory cytokines (MIP-1α, MIP-1β, MCP-1, IL-12, IL-1β, TNF-α, and IL-6 in infected macrophages with *L*. *tropica* amastigotes treated with 0.1 μM of Imiquimod or EAPB0503 for 10h. (B) RT-PCR detection of iNOS in infected macrophages with *L*. *tropica* amastigotes, treated with 0.1 μM of Imiquimod or EAPB0503 for 10h. iNOS percentage of expression was normalized to GAPDH. Results are expressed as percentage of untreated control (±) SD. (C) ELISA showing the secretion level of and anti-inflammatory cytokines (IL-10 and IL-4) upon treatment of *L*. *tropica* infected macrophages with 0.1 μM of Imiquimod or EAPB0503 for 10h. Results are expressed as percentage of untreated control (±) SD and are representative of at least three independent experiments. The t-test was performed to validate significance. *, ** and *** indicate p values ≤ 0.05; 0.01 and 0.001, respectively. P-values less than 0.05 were considered significant.

We investigated the expression of pro- and anti-inflammatory mediators after treatment. Macrophage Inflammatory Proteins (MIP-1α and β) and Monocyte Chemoattractant Protein (MCP-1) levels increased upon treatment with both drugs (Fig 3A). The secreted levels of depicted pro-inflammatory cytokines namely Interleukin-12 (IL-12), IL-1β, TNF-α and IL-6, important in CL clearance [24], were increased upon treatment with Imiquimod or EAPB0503 (Fig 3A).

Macrophage-derived nitric oxide (NO) is effective against microbes, and synthesized by Nitric Oxide Synthase (i-NOS). i-NOS is induced in response to pro-inflammatory cytokines [25] and, in CL-infected macrophages, iNOS is protective against *L*. *major* [26]. Both drugs increased i-NOS transcripts in macrophages infected with either *L*. *tropica* or *L*. *major* strains, with EAPB0503 inducing a 5-fold higher expression (Fig 3B, S2B Fig respectively). This presumably leads to higher NO production, hence enhanced leishmanicidal activity.

In CL, pro-inflammatory cytokines are linked to resistance against leishmaniasis; whereas anti-inflammatory cytokines relate to disease progression [27]. We examined the secretion levels of two depicted anti-inflammatory cytokines, IL-10 and IL-4 after treatment with either drugs. In comparison to non-treated *L*. *tropica* infected macrophages, secretion levels of IL-10 and IL-4 decreased by around 4 folds after treatment with Imiquimod (Fig 3C). More interestingly, treatment with EAPB0503 showed a significant decrease by 4 and 15 folds of IL-10 and IL-4 respectively and as compared to non-treated infected macrophages (Fig 3C).

Altogether, our results show that NF-κB activation by Imiquimod and EAPB0503 induces secretion of pro-inflammatory cytokines. This leads to i-NOS upregulation, presumably leading to NO production and leishmanicidal activity. In addition, and concomitantly with the upregulation of pro-inflammatory cytokines, a decrease in the anti-inflammatory cytokines is obtained, diminishing macrophage susceptibility to *L*. *tropica* infection, and triggering the leishmanicidal effect of the tested drugs.

### EAPB0503 exhibited a higher efficacy on freshly isolated *L*. *tropica* from patients’ biopsies

**Fig 4:**
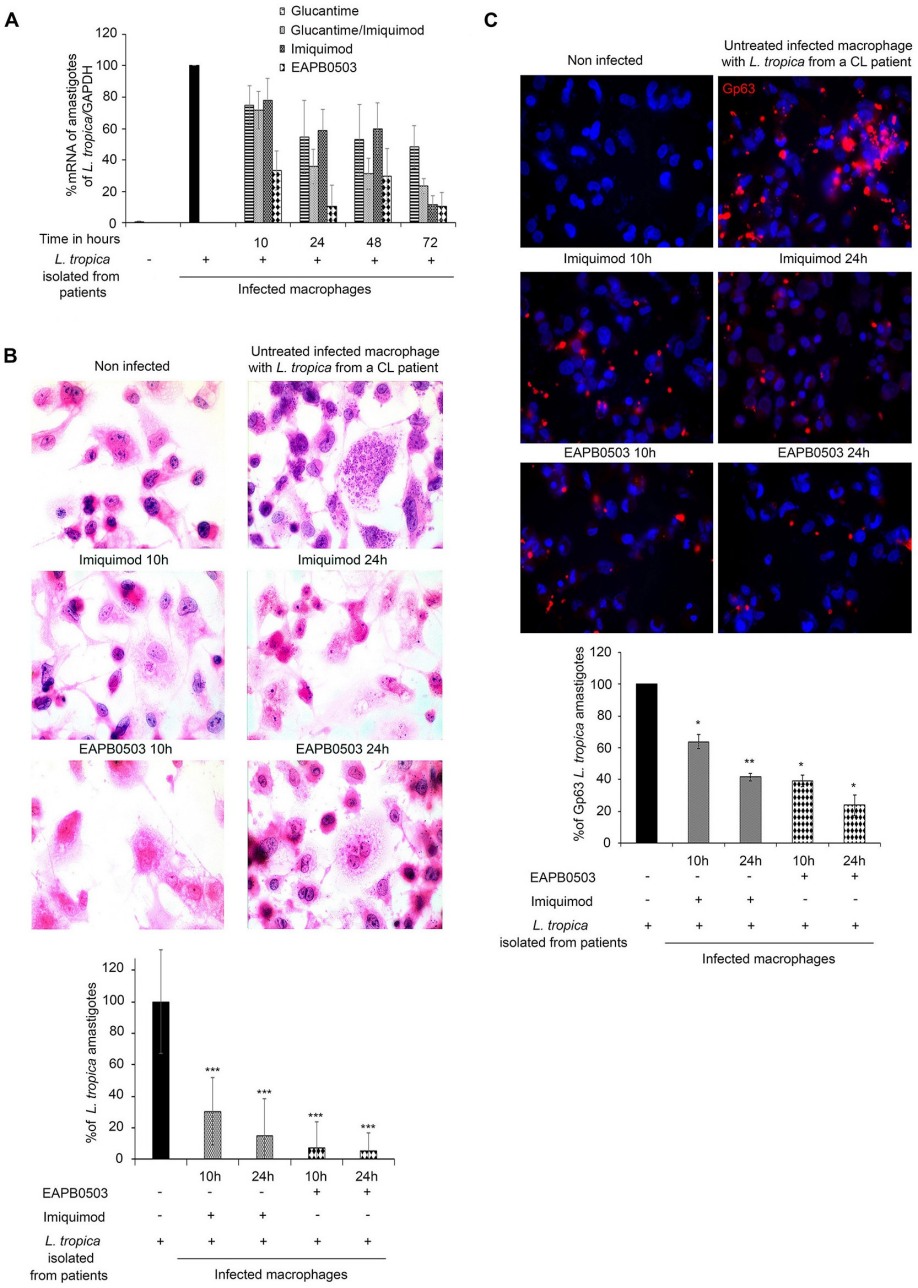
EAPB0503 exhibits a higher efficacy on freshly isolated *L*. *tropica* from patients’ biopsies. (A) RT- PCR detection of infected macrophages with patients’ derived *L*. *tropica* amastigotes treated with 0.1 μM of Imiquimod or EAPB0503 for 10 or 24h, compared to 100 μg/mL of Glucantime alone or combined to 0.1 μM of Imiquimod. Percentage of expression of amastigotes was normalized to GAPDH. Results are expressed as percentage of untreated control (±) SD and are representative of three independent experiments. (B) H&E staining on untreated or treated macrophages infected with patients’ derived *L*. *tropica* amastigotes with 0.1 μM of Imiquimod or EAPB0503 for 10 or 24h. The results depict one representative patient. Similar results were obtained on the remaining two patients. (C) Confocal microscopy on patients’ derived *L*. *tropica* infected macrophages treated with 0.1 μM of Imiquimod or EAPB0503 for 10 or 24h. The Gp63 surface parasite was stained with an anti-Gp63 antibody (red), and nuclei were stained with Hoechst 33342 (blue). Images represent Z sections. Graphs show quantification of Gp63 (Blind count) as averages of one Z section/cell from 50 different cells of 2 independent experiments on two different patients’ derived amastigotes.

To eliminate the potential doubt due to the susceptibility of cultured *L*. *tropica* and *L*. *major* strains to our tested treatments (e.g genetic drift and less virulent strains after long term culture), we investigated the effect of Imiquimod and its analog EAPB0503 on freshly isolated parasites from untreated patients’ biopsies. The infection with *L*. *tropica* was confirmed in all used patients by PCR and Restriction Fragment Length Polymorphism (RFLP) (S3A and S3B Fig respectively). Both drugs inhibit amastigote replication in a time dependent manner. As a control of potency, we used Glucantime, alone or combined with Imiquimod, since these drugs were clinically used in the treatment of CL [15, 16, 28]. Upon treatment with Imiquimod, amastigotes transcription levels decreased to reach around 10% at 72h post-treatment as compared to 60% upon treatment with Glucantime alone, or around 25% upon treatment with Glucantime combined to Imiquimod (Fig 4A). Treatment with EAPB0503 induced a more prominent effect where, *L*. *tropica* amastigote transcripts decreased by 70 and 90%, at 10 and 24h post-treatment respectively (Fig 4A). This effect was identical at 72h post-treatment (Fig 4A). Interestingly, the effect of EAPB0503 was more profound than Glucantime alone, Imiquimod alone or Glucantime combined to Imiquimod at all tested time points, and starting 10h post-treatment (Fig 4A). We then assessed the effect of both drugs on amastigotes histologically (Fig 4B) and by immunofluorescence confocal microscopy (Fig 4C). Our results were very consistent with the transcript data with less amastigotes detected upon treatment. Consistently, and using the *Leishmania* Glycoprotein Gp63 marker for quantification of amastigotes [29], Imiquimod treatment led to a decrease in amastigotes percentage reaching 60% at 10h and 35% at 24h post-treatment (Fig 4C). More interestingly, EAPB0503 induced a more prominent decrease in amastigotes number, to 40% after 10h of treatment, and to 25% after 24h of treatment (Fig 4C). Altogether, these data show that Imiquimod and mostly EAPB0503 are highly active at the low dose of 0.1 μM and as early as 10h on patients’ derived *L*. *tropica* stages, confirming the obtained results on *in vitro* cultured strains.

**Fig 5:**
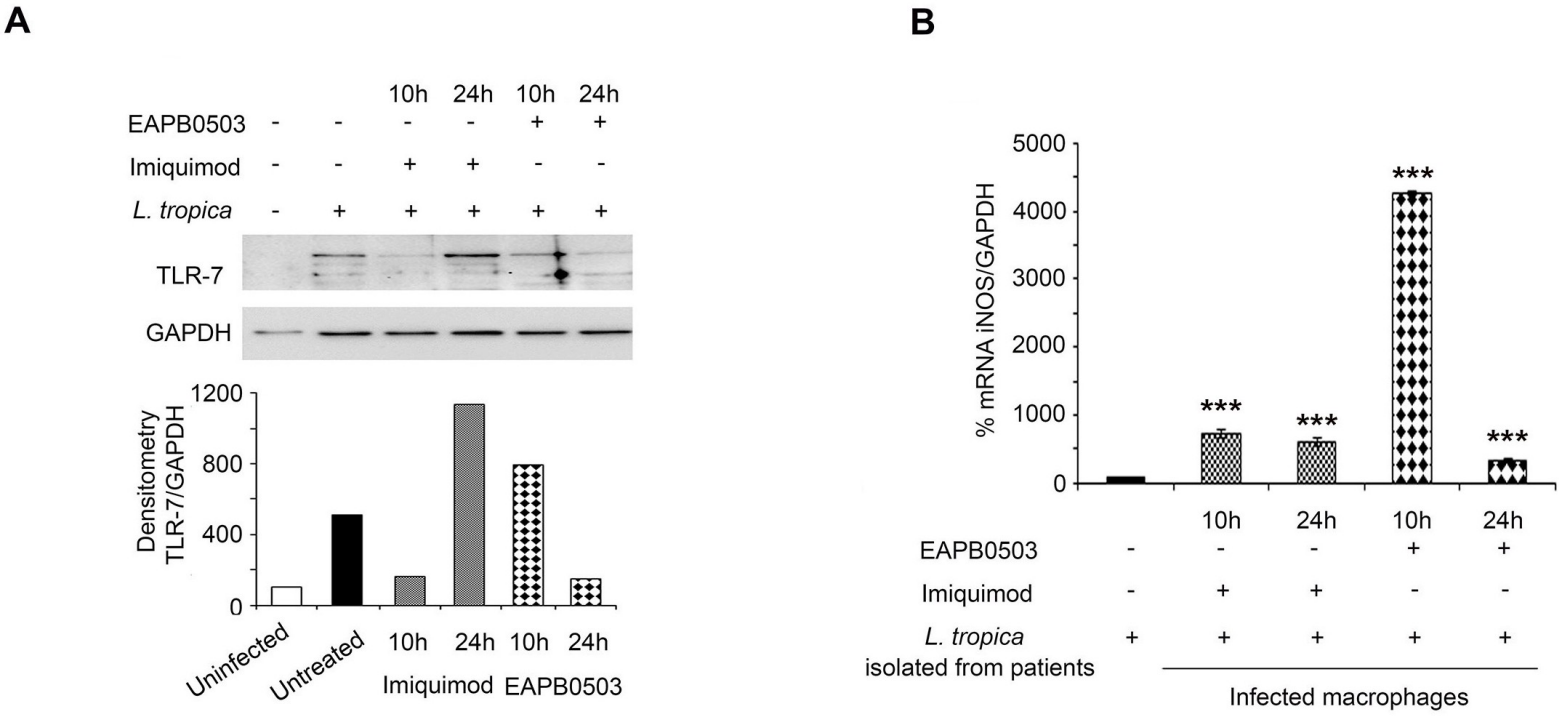
Imiquimod and EAPB0503 trigger an upregulation of TLR-7 and iNOS expression in patients’ derived *L*. *tropica* infected macrophages. (A) Western blot analysis for TLR-7 on patients’ derived *L*. *tropica* infected macrophages (from one patient) treated with 0.1 μM of Imiquimod or EAPB0503 for 10 and 24h. (B) RT- PCR detection of iNOS in infected macrophages with patients’ isolated *L*. *tropica* amastigotes upon treatment with 0.1 μM of Imiquimod or EAPB0503 for 10 and 24h. Percentage of expression of amastigotes was normalized to GAPDH. Results are expressed as percentage of untreated control (±) SD and are representative of three independent experiments. The t-test was performed to validate significance. *, ** and *** indicate p values ≤ 0.05; 0.01 and 0.001, respectively. P-values less than 0.05 were considered significant.

The mechanism of action of either drugs on *L*. *tropica* obtained from patients, was evaluated for TLR7 protein expression and showed an increase after treatment, in comparison to uninfected or untreated infected macrophages. Consistently with the cultured strain, Imiquimod induced the highest TLR7 protein levels (Fig 5A). Moreover, i-NOS transcript levels were increased reaching the highest levels after 10h treatment with EAPB0503 (Fig 5B). These results indicate a similar mode of action of both drugs on freshly isolated parasites from patients’ biopsies and confirm the higher potency of EAPB0503 against CL.

## Discussion

CL is one of the most common neglected tropical diseases worldwide. Globally, the annual incidence of CL is estimated to be 0.7 to 1.2 million new cases per year. This disease is still endemic in many countries [30]. In the Eastern Mediterranean, Syria presents with the highest number of CL cases [31]. The Syrian conflict exacerbated the spread of the infection to the surrounding areas. In Lebanon, 85% of infected Syrian refugees were diagnosed with *L*. *tropica* whilst the remaining 15% were infected with *L*. *major* [5].

Pentavalent antimony compounds remain the treatment choice for CL. However, these compounds associate with high cost, poor availability, drug resistance and systemic toxicity [32]. We focused on testing novel drugs’ efficacy on *L*. *tropica* and *L*. *major*. Imiquimod activates macrophages [15], the main host cells for *Leishmania* replication. In CL, Imiquimod was mainly tested against *L*. *major* amastigote [33]. In Imiquimod treated mice infected with *L*. *major*, an increased protection was obtained [34]. In CL patients, Imiquimod combined to Glucantime induced a high healing rate in refractory patients [13, 16, 34]. We showed that both drugs affected amastigotes. Conversely, EAPB0503 was more potent on *L*. *tropica* strain [4, 5]. Imiquimod acts *via* binding TLR7, leading to the activation of the NF-κB pathway. Imiquimod protective effect, on *L*. *major* infected mice, was coupled with the induction of NO synthesis [26, 35]. Consistent with the published data, but on *L*. *tropica* strain, we showed that Imiquimod and EAPB0503 upregulated TLR7 expression. Nonetheless, the highest induction was obtained upon Imiquimod treatment. This finding suggests that EAPB0503 may partially act *via* TLR7, or through other TLRs. The canonical NF-κB pathway was activated by both drugs, leading to increased secretion levels of pro-inflammatory cytokines. MIP-1α and MIP-1β, involved in resistance against infections [36] were both secreted at higher levels upon treatment with either drugs. Consistently with the known role of MIP-1α and MIP-1β in recruiting other cytokines such as TNF-α and IL-6 [37], levels of secretion for these two cytokines were also increased. TNF-α increased secretion levels were consistent with its protective role against CL [38].

Previous studies have shown that MCP-1 is highly expressed in lesions of patients with self-healing localized cutaneous leishmaniasis whereas it is scarce in those of chronic diffuse cutaneous leishmaniasis [39]. This suggests its role in the parasites elimination *via* induction of reactive oxygen intermediates (ROI). Our results showed that MCP-1 levels increased upon treatment with either drugs, but more importantly with EAPB0503, presumably explaining its higher potency. However, the potential involvement of ROIs on the clearance of treated parasites remains to be elucidated.

We also succeeded to test the activity of Imiquimod and EAPB0503 on freshly isolated *L*. *tropica* from skin lesions of CL patients. We confirmed the results obtained on cultured strains, thus eliminating any potential doubt about a lower virulence or a genetic drift obtained from long term cultures. Moreover, EAPB0503 showed a better anti-leishmanicidal activity than the clinically used Glucantime, whether alone or combined to Imiquimod [15, 16, 28]. These results highlight the promising potency of EAPB0503 for CL treatment.

Nitric Oxide production by activated macrophages is known to play a major role in fighting against infections [40], including *Leishmania* [41]. Inhibition of i-NOS reduced *L*. *infantum* burden in human macrophages [42]. In addition, the increase of i-NOS and NO generation in response to IFN-γ and TNF-α is crucial to control CL [43]. We checked for i-NOS transcripts in treated macrophages infected with either *L*. *major* or patients’ derived *L*. *tropica* and showed an important increase with either drugs. Interestingly, the highest levels were obtained upon EAPB0503 treatment, presumably explaining its higher leishmanicidal efficacy.

TLRs are important pattern recognition receptors expressed abundantly on macrophages. Early studies concluded that TLR2, TLR4, and TLR9, are involved in the recognition of *L*. *major* and that TLR2 ligands play a protective immune role against Leishmaniasis [44]. However, recent studies on C57BL/6 mice deficient in either TLR2, 4, or 9, showed that only TLR9^-/-^ mice are more susceptible to *L*. *major* infection, indicating TLR2 and TLR4 related immunity to murine leishmaniasis requires re-evaluation [45]. In this study, we confirmed that Imiquimod displays its anti-amastigote activity *via* TLR7 upregulation, leading to NF-κB activation and pro-inflammatory cytokine production. EAPB0503 effect on TLR7 was less prominent. Whether EAPB0503 acts *via* any of the important TLRs in CL or not, requires further investigation.

Collectively, our results did not only show a promising efficacy of a new compound, EAPB0503 against CL, but also highlighted the mechanism of action through which Imiquimod and its analog acted against the aggressive *L*. *tropica* strain. We also described the molecular mechanisms of these drugs against amastigotes highlighting the importance of immune-modulatory therapy against CL.

## Materials and methods

### Parasite culture

*Leishmania major* (MHOM/MA/81/LEM265 and MMER/MA/81/LEM309) and *Leishmania tropica* (MHOM/LB/76/LEM61, MRAT/IQ/72/ADHANIS1) were purchased from the CRHU “Montpellier”. Parasites were maintained in RPMI1640 (Lonza) supplemented with 10% Fetal Bovine Serum (FBS), 100IU/ml streptomycin/penicillin (Sigma).

### Test agents

Imiquimod was purchased from Molekula (Wessex House) and EAPB0503 was synthesized using microwave-assisted chemistry as described by Khier et al. [46]. Drugs were prepared as a 0.1 M stock in dimethylsulfoxide (DMSO) and stored at -80°C. Glucantime (1.5g/5ml) was used at the final concentration of 100 μg/mL. Working solutions of 0.1 μM were freshly prepared in culture media.

### Macrophage culture and treatment

Human monocytic THP-1 cells (American Type Culture Collection (ATCC TIB-202), Manassas, VA) were grown in RPMI1640 medium with L-Glutamine, 25 mM Hepes (Lonza), supplemented with 10% of fetal bovine serum (FBS), 1% penicillin-streptomycin, 1% kanamycin and 1% glutamine (Invitrogen). 1 million THP-1 cells were differentiated into macrophages, using 50 ng/mL of phorbol 12-myristate 13-acetate (PMA, Sigma) overnight. Following their adherence, differentiated macrophages were then activated with 1 μg/mL of LPS for 4h, then infected with *L*. *major* or *L*. *tropica* at the ratio of 5 parasites/macrophage, and incubated for 24h at 37 ᴼC. Non-internalized promastigotes were removed by two gentle washes with PBS.

### Isolation of fresh *L*. *tropica* promastigotes from biopsies of CL patients

Punch biopsies (4 mm of diameter) from three CL patients were performed in 2016, and incubated in sterile physiological serum, supplemented with Penicilline G (100 IU/ml). Specimens were incubated in a semi-solid culture media (10g agar, 3g NaCl, 500 mL water). 3 weeks later, promastigotes were transferred to liquid medium.

### Anti-amastigote activity

Macrophages infected with *L*. *major*, *L*. *tropica*, or patients’ derived *L*. *tropica* parasites were treated with Imiquimod and EAPB0503 (0.01 μM, 0.05 μM, 0.1 μM, 0.5 μM and 1 μM) for 24h. Total RNA was extracted using Trizol (Qiagen). cDNA synthesis was performed using a Revert Aid First cDNA synthesis Kit (#K1622-Thermo Scientific). Syber green qRT PCR was performed using the BIORAD-CFX96 machine. Primers for the housekeeping Glyceraldehyde-3-Phosphate dehydrogenase GAPDH, and i-NOS are listed in Table 1. Primers for amastigotes detection target the minicircle kinetoplast DNA (kDNA) (Table 1). Percentage of expression was calculated according to Livac method [47].

**Table 1:** List of primers.

Primer	Sequence 5’→3’	References
GAPDH Forward Primer	5’-CATggCCTTCCgTgTTCCTA-3’	[48]
GAPDH Reverse Primer	5’-CCTgCTTCACCACCTTCTTgAT-3’	[48]
Kinetoplast Forward Primer	5’-CCTATTTTACACCAACCCCCAGT-3’	[49]
Kinetoplast Reverse Primer	5’- GGGTAGGGGCGTTCTGCGAAA -3’	[49]
i-NOS Forward primer	5′-GGGAGCCAGAGCAGTACAAG-3′	[43]
i-NOS Reverse primer	5′-GGCTGGACTTCTCACTCTGC-3′	[43]
*Leishmania* L5.8S Forward	5’-TGATACCACTTATCGCACTT-3’	[49]
*Leishmania* Internal Transcribed spacer (LITS)-Reverse	5’-CTGGATCATTTTCCGATG-3’	[49]

### Enzyme-linked immunosorbent assay (ELISA)

Supernatants of infected macrophages in presence or absence of either drugs were collected 10h and 24h after treatment, and ELISA was performed using Multi-Analyte ELISArray Kit (Qiagen) according to the manufacturer’s instructions. Briefly, supernatants of *L*. *tropica* infected macrophages (untreated or treated with 0.1 μM of Imiquimod or EAPB0503) were collected. Supernatants were spun for 10 min at 1000g and transferred to new Eppendorf tubes, and diluted using a specific cocktail of antigens (IL-12, IL-1β, IL-6, and TNF-α, MIP-1α, MIP-1β, MCP-1, IL-10 and IL-4) provided by the kit (Qiagen). Samples were then loaded in the coated ELISA plaque, and were incubated for 2 hours. 3 washes were performed, and the detection antibody was added and incubated for 2 hours. Then, Avidin-HRP was added for 30 min, and 4 washes were performed. Development solution was then added in dark and kept for 15 min, before addition of the stop solution. The secreted levels of the following cytokines and chemokines were then assessed. The optic density (O.D) was determined at 450 and 570 nm and calculated according to the standard values of a positive control provided by the kit.

### Immunofluorescence and confocal microscopy

For Immunofluorescence assay, p6 well plates were seeded with activated macrophages infected with *L*. *tropica* (5p/c) for 24h and treated with Imiquimod or EAPB0503 for 10 or 24h. At these time points, coverslips were fixed in 4% paraformaldehyde for 20 minutes. Permeabilization was performed in Triton (0.2%) for 10 minutes. Following one PBS wash, blocking for 30 min with PBS-10% FBS was performed. Primary antibody directed against the NF-κB p65 subunit (Santa Cruz, Sc-8008) was used at the dilution of 1:50. For *Leishmania* parasite staining inside macrophages, an anti-Gp63 (LifeSpan BioSciences, LS-C58984) was used at the dilution 1:50. Anti-mouse secondary antibodies (Abcam, ab150116) were used at the concentration of 1:100. Staining of nuclei was performed using 1 μg/mL of Hoechst 33342, trihydrochloride trihydrate solution (Invitrogen, H33342) for 5 min and then coverslips were mounted on slides using a Prolong Anti-fade kit (Invitrogen, P36930). Z-section images were acquired by confocal microscopy using a Zeiss LSM 710 confocal microscope (Zeiss, Germany) and all images were analyzed using Zeiss LSM 710 software.

### Hematoxylin and eosin stain

H&E staining was performed as described by Grosset et al., 2017 [50]. Briefly, hematoxylin (Fisher Scientific, Canada) was added on cells, and a counterstaining for 30 seconds was performed followed by a water rinse for 5 minutes. Slides were then dipped in 50% (vol/vol) alcoholic eosin Y solution (Leica Microsystems, Canada) then rinsed in ethanol before slide mounting.

### Giemsa staining

Giemsa staining was performed using Ral 555 Kit (RAL Diagnostics). Briefly, cells were fixed with methanol for 1 min, stained with solution 2 for 40 seconds then with Solution 3 for 25 seconds as per the manufacturer. Cells were then mounted using Prolong Anti-fade (Invitrogen, P36930).

### Western blot analysis

Activated macrophages infected with *L*. *tropica* (5p/c) for 24h were treated with 0.1 μM of Imiquimod or EAPB0503 for 10 or 24h. Cells were scrapped, washed with PBS, and pellets were re-suspended in 1x Laemmli buffer. Following denaturation, samples were run on 10% polyacrylamide gels. Proteins were then transferred to nitrocellulose membranes (BIO RAD Cat# 162–0112) at 30V overnight using a BioRad transfer unit. To verify the protein transfer, nitrocellulose membranes were stained with Ponceau Red. Blocking was performed for 1h in 5% of Bovine Albumin serum (BSA) in wash buffer and probed with specific primary antibodies against TLR7 (sc- 57463 Santa Cruz Biotechnology, 1:100), NF-κB p65 (sc-8008; Santa Cruz Biotechnology, 1:250), or p52 (sc-7386, Santa Cruz Biotechnology, 1:250). Equal loading was tested following probing with the anti- GAPDH antibody (MAB5476; abnova, 1:20 000). Nitrocellulose membranes were then washed three times with wash buffer for 5 minutes each, before incubation with the appropriate anti-mouse secondary antibody conjugated to Horseradish peroxidase (HRP) (m-IgGk BP-HRP, Santa Cruz, sc-516102, 1:5000). Bands were visualized by autoradiography, following incubation with luminol chemiluminescent substrate (Bio-Rad, Cat# 170–5061).

### Internal Transcribed Spacer-1 (ITS1)-PCR and Restriction Fragment Length Polymorphism (RFLP) analysis of amplified ITS1-PCR amplicons

DNA extraction from patient’s biopsies and PCR were performed as previously described using ITS-1 primers (Table 1) [49]. Following PCR amplification, 10 μl of the remaining volume of the amplicon was digested with 2 μl MnII enzyme, in 2 μl in 10x Buffer G (Fermentas Life Sciences, Thermo Fisher Scientific) and 18μl of nuclease-free water. Digestion was carried out using the TS100 thermal cycler (Biorad) by incubating for 6h at 37°C, followed by enzyme inactivation for 20 minutes at 65°C.

25 μl of the digestion products were electrophoresed on 1.5% agarose gels at 100 V in 1× TBE buffer (0.04 M Tris-acetate and 1 mM EDTA, pH 8.0) for 30 minutes. The restriction fragments were visualized under UV light. Images were then captured using the GelDoc-IT TM Imaging System and RFLP patterns interpreted for sub-speciation. *Leishmania tropica* is characterized by the presence of two bands of 300 base pairs (bp) and 50bp respectively.

### Ethics statement

Sample collection was approved by the Institutional Review Board of the American University of Beirut (PALK.IK.01). A parent of each of the three children participants provided written informed consent on the child’s behalf before sample collection.

### Statistical analysis

Continuous variables were analyzed by the unpaired Student’s t test. P value was determined and values for p < 0.05 were considered as significant.

## Supporting information

S1 Fig DMSO alone has minimal effect on *L*. *tropica* infected macrophages. RT- PCR detection of uninfected macrophages, or infected macrophages with patients’ derived *L*. *tropica* amastigotes either untreated or treated with 0.1% of DMSO for 24h.
(JPG)

S2 Fig Imiquimod and EAPB0503 trigger an increase of TLR-7 and i-NOS expression in *L*. *major* infected macrophages. (A) Western blot analysis for TLR-7 in *L*. *major* infected macrophages treated with 0.1 μM of Imiquimod or EAPB0503 for 10 and 24h. The results depict one representative experiment among three independent ones. Densitometry was performed using Image Lab software (Biorad). Results shown represent the average of quantification of three independent experiments. (B) RT- PCR detection of i-NOS in infected macrophages with *L*. *major* amastigotes upon treatment with 0.1 μM of Imiquimod or EAPB0503 for 10 and 24h. Percentage of expression of amastigotes was normalized to GAPDH. Results are expressed as percentage of untreated control (±) SD and are representative of three independent experiments. The t-test was performed to validate significance. *, ** and *** indicate p values ≤ 0.05; 0.01 and 0.001, respectively. P-values less than 0.05 were considered significant.
(JPG)

S3 Fig The three CL patients are infected with *L*. *tropica*. (A) Gel electrophoresis for the Internal Transcribed Spacer-1 (ITS-1) amplicon of one patient. A band of 300 bp is an indicator of CL infection. (B) Gel electrophoresis after Restriction Fragment Length Polymorphism (RFLP) using MnII restriction enzyme, on one CL patient. The results depict one representative patient. Similar results were obtained on the remaining two patients.
(JPG)
