# Supplementary figures and images for: EAPB0503: An Imiquimod analog with potent in vitro activity against cutaneous leishmaniasis caused by Leishmania major and Leishmania tropica
Source: PLoS Negl Trop Dis. 2018 Nov 21;12(11):e0006854. doi: 10.1371/journal.pntd.0006854 (PMC6248897; doi:10.1371/journal.pntd.0006854)

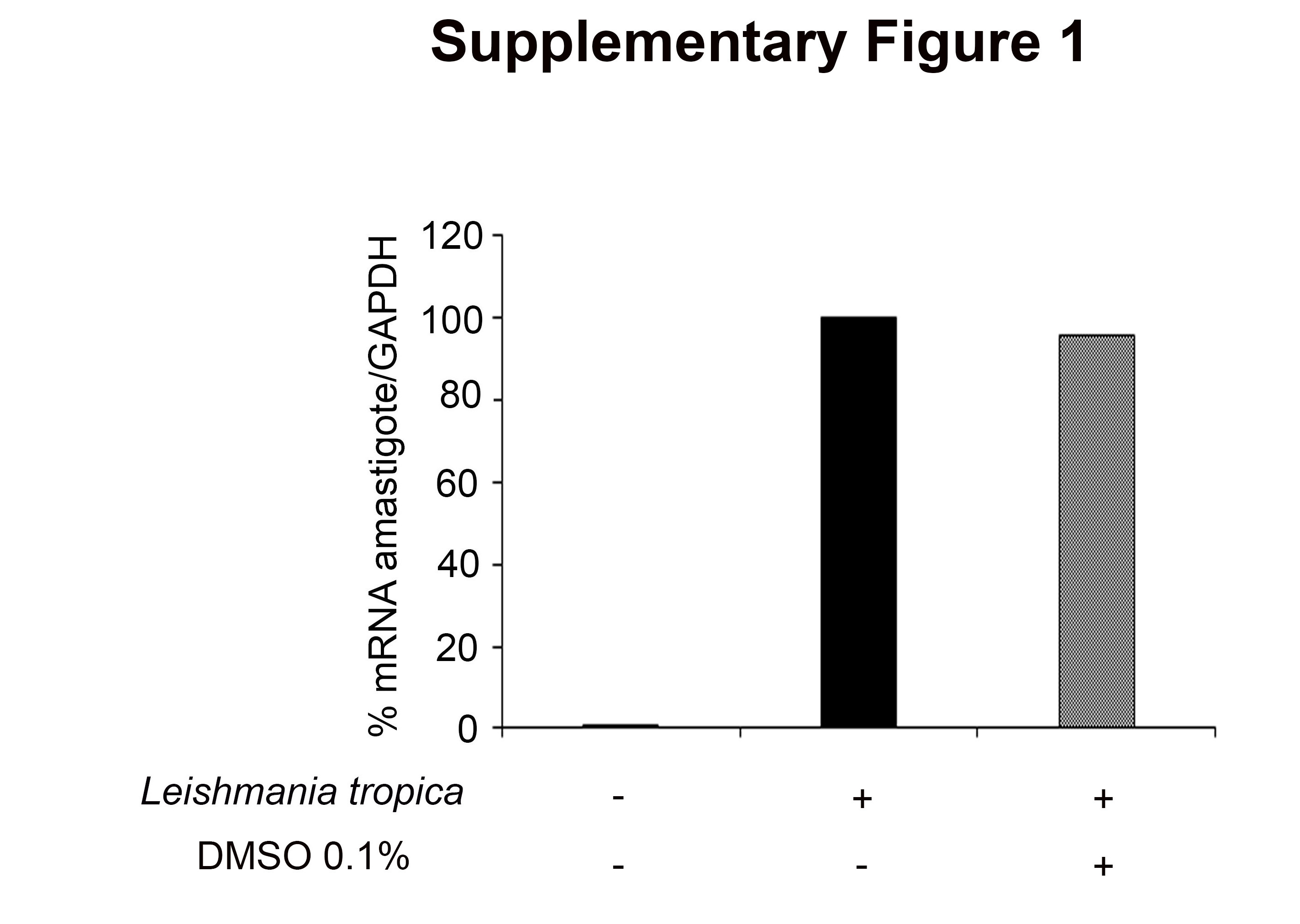

Supplement: S1 Fig — RT- PCR detection of uninfected macrophages, or infected macrophages with patients’ derived L. tropica amastigotes either untreated or treated with 0.1% of DMSO for 24h. (JPG) [file pntd.0006854.s001.jpg]

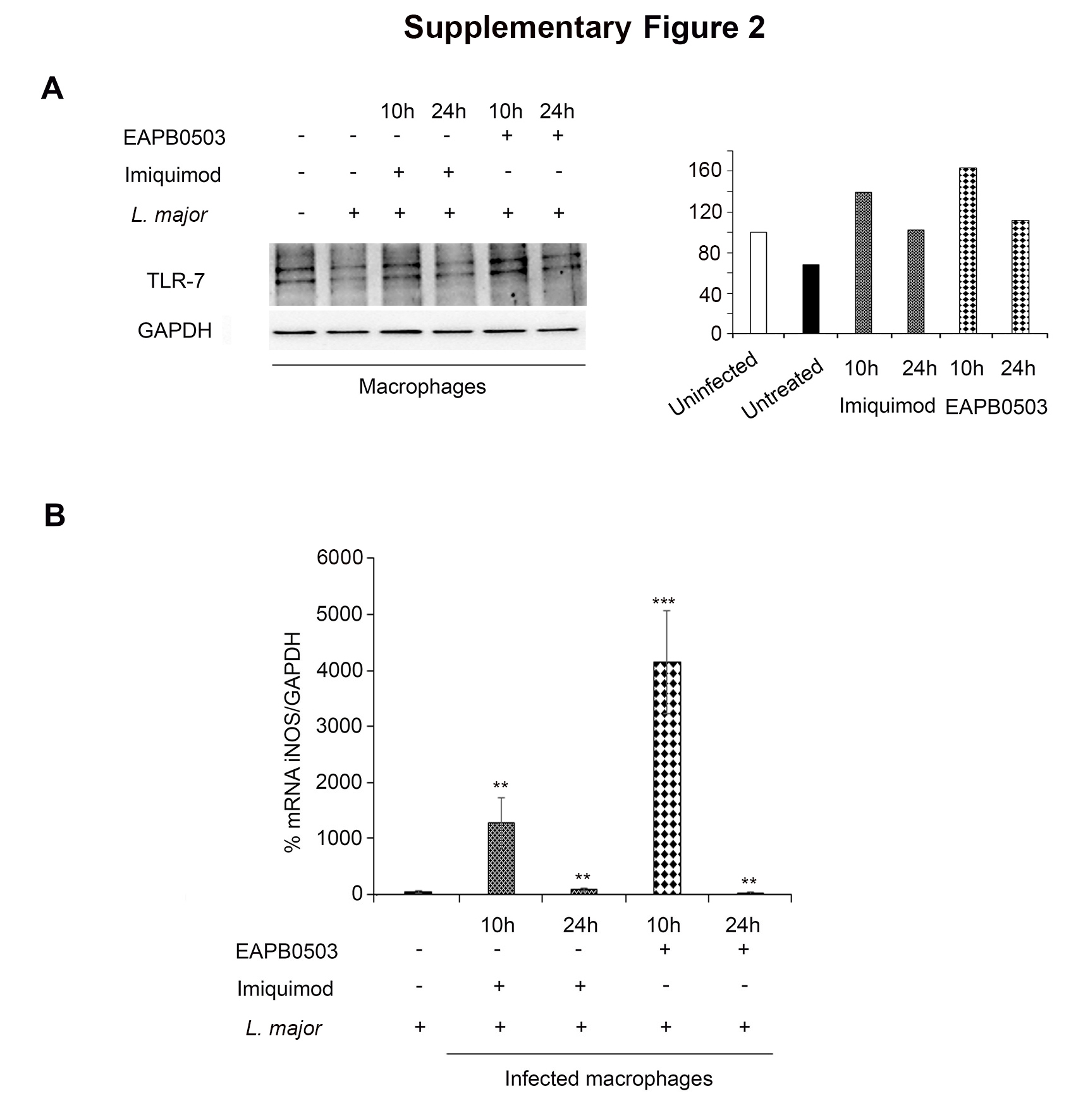

Supplement: S2 Fig — (A) Western blot analysis for TLR-7 in L. major infected macrophages treated with 0.1 μM of Imiquimod or EAPB0503 for 10 and 24h. The results depict one representative experiment among three independent ones. Densitometry was performed using Image Lab software (Biorad). Results shown represent the average of quantification of three independent experiments. (B) RT- PCR detection of i-NOS in infected macrophages with L. major amastigotes upon treatment with 0.1 μM of Imiquimod or EAPB0503 for 10 and 24h. Percentage of expression of amastigotes was normalized to GAPDH. Results are expressed as percentage of untreated control (±) SD and are representative of three independent experiments. The t-test was performed to validate significance. *, ** and *** indicate p values ≤ 0.05; 0.01 and 0.001, respectively. P-values less than 0.05 were considered significant. (JPG) [file pntd.0006854.s002.jpg]

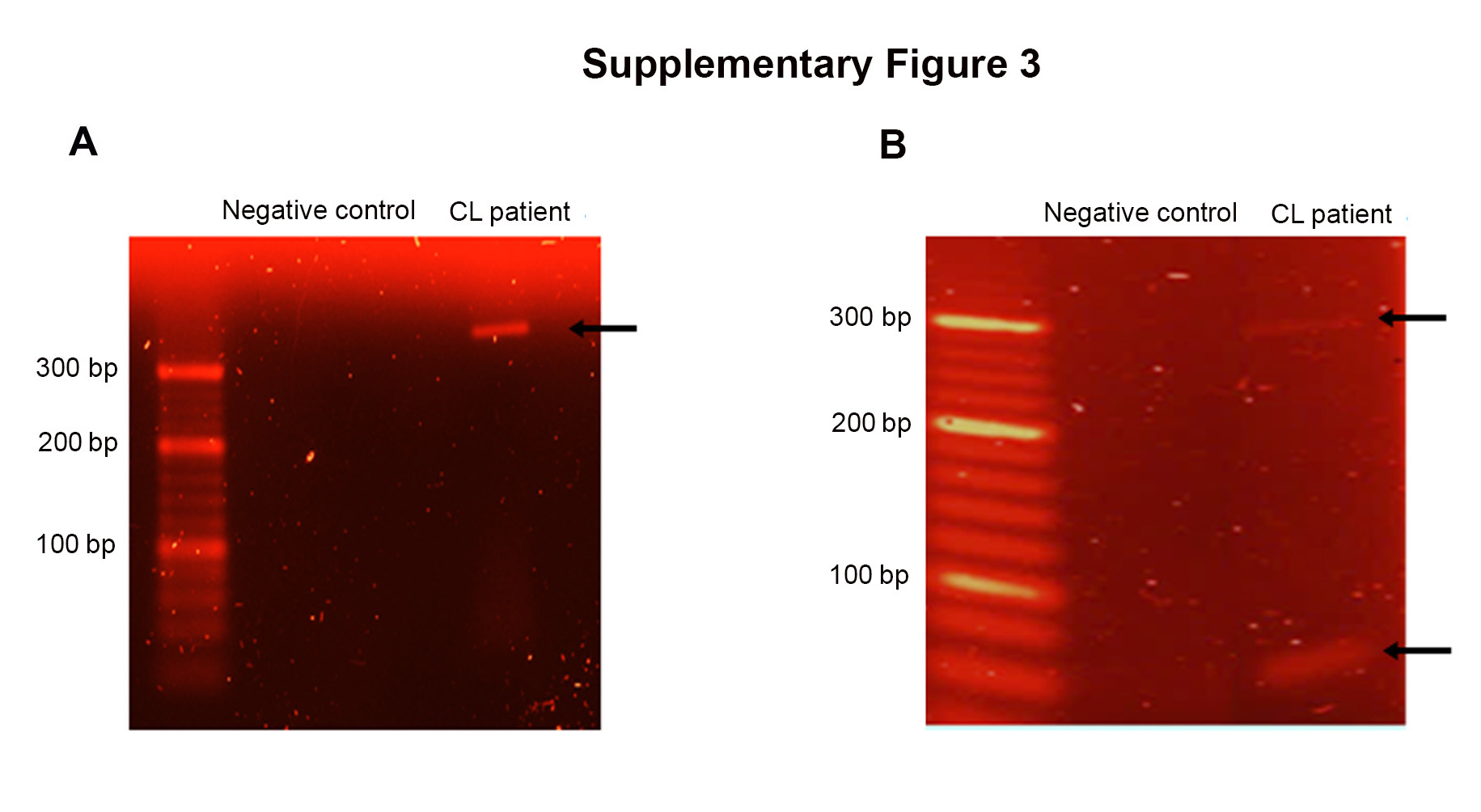

Supplement: S3 Fig — (A) Gel electrophoresis for the Internal Transcribed Spacer-1 (ITS-1) amplicon of one patient. A band of 300 bp is an indicator of CL infection. (B) Gel electrophoresis after Restriction Fragment Length Polymorphism (RFLP) using MnII restriction enzyme, on one CL patient. The results depict one representative patient. Similar results were obtained on the remaining two patients. (JPG) [file pntd.0006854.s003.jpg]
